# Gender associated effects of the ethanolic extracts of Chinese propolis on the hepatic transcriptome in ethanol-treated mice

**DOI:** 10.22038/ijbms.2019.37348.8886

**Published:** 2019-10

**Authors:** Manhong Ye, Mengting Xu, Mengmeng Ding, Chao Ji, Jian Ji, Fubiao Ji, Wanhong Wei, Shengmei Yang, Bin Zhou

**Affiliations:** 1 College of Bioscience and Biotechnology, Yangzhou University, Yangzhou 225009, Jiangsu Province, China; 2 Fubiao Biotech Co, Ltd, Huai-an 211799, Jiangsu Province, China; 3 Joint International Research Laboratory of Agricultural & Agri-Product Safety, Yangzhou University, Yangzhou 225009, Jiangsu Province, China; 4 College of Animal Science and Technology, Yangzhou University, Yangzhou 225009, Jiangsu Province, China

**Keywords:** Alcoholic, Chinese propolis, Fatty liver, Inbred C57BL, Lipid metabolism, Mice

## Abstract

**Objective(s):**

The current study investigated the potential hepatoprotective effects of the ethanolic extracts of Chinese propolis (EECP) on ethanol-induced fatty liver in mice.

**Materials and Methods:**

C57BL/6J mice were orally gavaged with 50% ethanol alone or co-administrated with EECP at the dose of 0.2 ml/kg bodyweight for eight weeks. The dose for ethanol was 6 ml/kg bodyweight for the first two experimental weeks, and then increased to 8, 10, and 12 ml/kg bodyweight every two experimental weeks. Alterations in the hepatic transcriptome due to concomitant administration of EECP were investigated using RNA-Seq technique.

**Results:**

Our results showed that the main EECP-responsive genes were involved in lipid syntheses, which were significantly down-regulated in both female and male mice co-administrated with EECP. In female mice, these differentially expressed genes (DEGs) were mainly associated with fatty acid biosynthesis. While in male mice, these DEGs were mainly involved in the steroid metabolic process and cholesterol biosynthetic process. Despite the sex-associated responses in lipid metabolism, EECP also exerted other beneficial effects in female mice through modulation of the cytokine-cytokine receptor interaction pathway that helped explaining its hepato-protective effectiveness.

**Conclusion:**

Our findings indicated that the mechanism regarding the hepato-protective effects of EECP was gender-dependent, which is worthy of further investigation during the development of therapeutic interventions using EECP to reduce the adverse influences of ethanol.

## Introduction

Alcohol consumptions are prominent risk factors worldwide for the development of alcohol-attributable liver diseases, which have contributed markedly to the global burden of morbidity and mortality (1). Despite the profound health impact of alcoholic liver disease (ALD), only limited interventions are available, which include nutritional support, treatments with tumor necrosis factor-α (2), corticosteroids alone or in association with pentoxifylline or N-acetylcysteine (3). Therefore, there is an increasing demand to explore (non)pharmaceutical interventions to address this important public health issue.

Propolis is a natural bee-metabolized resinous substance collected by honeybees from bud secretions, leaves, and exudates of different plants. It has been widely used in folk medicine and is receiving increased attention for its potential use as a dietary supplement and health promoter due to its various biological properties (4). Chinese propolis is characterized by its abundance in flavonoids, including chrysin, pinocembrin, pinobanksin-acetate, and galangin (5). Of which, chrysin has been demonstrated to exert global hepatoprotective effects in chemical- and drug-induced liver injuries, such as attenuating inflammation and liver fibrosis (6, 7), improving serum lipid profile, reducing oxidative stress and hepatotoxicity (8-10). Pinocembrin has been identified as a potential anti-fibrotic agent in the treatment of liver fibrosis (11). Pinobanksin possesses potential hepato-protective activity due to its antioxidant activity (12). As for galangin, it has exhibited hepato-protective effects in hepatitis (13), drug-induced liver failure (14), and liver fibrosis (15). Based on this evidence, we hypothesized that the ethanolic extracts of Chinese propolis (EECP), rich in these flavonoid components, will show hepato-protective effects on ALD.

Steatosis (fatty liver), characterized by the accumulation of triglycerides in hepatocytes, is the initial and reversible stage of ALD (16). Timely treatment of steatosis is the most direct approach to prevent ALD from progressing to more severe forms, such as alcoholic hepatitis, fibrosis, cirrhosis, and hepato-cellular carcinoma (17). Up till now, the influences, as well as the mechanisms regarding the hepato-protective effects of EECP on ALD, have not been elucidated. The present work was designed to investigate the alterations in the hepatic transcriptome in response to the supplementation of EECP in alcohol-treated mice with steatosis, unravel the possible mechanisms involved in the hepato-protective role mediated by EECP, and provide information for developing effective therapeutic approaches using propolis as dietary supplementation in the treatment of ALD.

## Materials and Methods

***Ethics statement***

All protocols in this study adhered to animal welfare procedures and were approved by the Animal Ethical and Welfare Committee of Yangzhou University, Yangzhou, Jiangsu Province, China. 

***Ethanolic extracts of Chinese propolis (EECP)***

EECP used in this study was provided by Rigao Bee Products Co. Ltd. (Huaian, China). Briefly, raw propolis was fully grounded at -18 ^°^C. Then, four volumes of 80% ethanol (v/w) were added into the ground powder and allowed to stand at room temperature for 24 hr with constant agitation in darkness. This extraction procedure was repeated thrice. Each time, the supernatant was collected, and fresh 80% ethanol was added into the residues. All ethanolic extractions were combined and filtered through filter paper to remove solid impurities. The filtrate was frozen at -20 ^°^C to get rid of the wax and then subjected to distillation using a rotary vacuum evaporator under reduced pressure to remove ethanol. Finally, the obtained ethanol-free extracts were lyophilized and stored at -20 ^°^C. The components in EECP detected by GC-MS were shown in supplemental Table 1. Before use, 30 gr EECP was dissolved in 100 ml of ethanol to the concentration of 30% (w/v).

***Animals and experimental design***

Twelve C57BL/6J mice (half male and half female), aged 8 weeks and weighing 20.59±3.12 g, were purchased from the Comparative Medical Centre of Yangzhou University, Yangzhou, Jiangsu Province, China (animal license, SYKX SU 2017-0001; ethnics license: 1403070), and maintained individually in plastic cages in an air-conditioned room (temperature, 22±3 ^°^C; humidity, 40–70%; 12 hr light: 12 hr dark cycle per day, 6:00–18:00). Animals had constant access to food and chlorinated tap water. They were acclimated to the laboratory for one week prior to dosing. 

There were two experimental groups in this study, group ET (treated with ethanol only) and group ET-EECP (treated with ethanol and co-administered with EECP). Each group contained three male mice and three female mice. The whole experiment lasted eight weeks. The procedure for the establishment of alcohol-induced steatosis in mice was performed according to Bai* et al. *(18). Mice in group ET were gavaged with 50% ethanol at the dose of 6 ml/kg bodyweight for the first two experimental weeks, and then increased to 8, 10, and 12 ml/kg bodyweight every two experimental weeks. The same regime was followed by animals in group ET-EECP except that 30% EECP was co-administrated with ethanol. The dose of 30% EECP was 0.2 ml/kg bodyweight. At the end of the experiment, mice were anesthetized by intraperitoneal injection of sodium pentobarbital (150 mg/kg). Liver samples were snap-frozen in liquid nitrogen, after being weighed, and then stored at -70 ^°^C. Relative liver weight (RLW) was recorded as the percentage of liver tissue weight to bodyweight (BW).

***Hepatic metabolites***

The hepatic levels of triglyceride (TG) and total cholesterol (TC) were determined using commercial kits from Nanjing Jiancheng Bioengineering Institute (Nanjing, China). Briefly, ultrasonic disruption was used to homogenize 100 mg of liver tissue in ice-cold ethanol (1:9), which was then centrifuged at 2,500 g for 10 min at 4 ^°^C. The supernatant was collected, stored on ice, and used for TG quantification using the Tissue Triglyceride Quantification Kit. For cholesterol quantification, 10 mg of liver tissue was extracted with 200 µl of chloroform in a microhomogenizer. The extract was centrifuged at 12,000 g for 10 min, and the organic phase was transferred to a new tube. Chloroform was removed by using a nitrogen blowing instrument. The dried lipids were re-dissolved in 200 µl of ethanol via vortexing. TC was quantified using the Total Cholesterol Assay Kit. 

***RNA isolation and sequencing the transcriptome***

The preparation and sequencing of RNA-Seq library were conducted by staff in Genepioneer Biotech Co, Ltd. (Nanjing, China). All kits were applied following the manufacturer’s recommendations. Briefly, total RNA was extracted from 12 individual liver samples using the RNeasy Mini Kit (Qiagen, Texas, USA) and then digested with DNase I to get rid of genomic DNA. The RNA quality was then assessed with an Agilent 2100 Bioanalyzer (Agilent Technologies, Santa Clara, CA, USA). RNA samples with integrity number larger than 8.7 were used to generate polyA-enriched mRNA libraries using the Illumina TruSeq RNA Sample Preparation Kit v2. Unique index codes were added to attribute sequences to each sample. The 12 libraries were then quantitated with Qubit 2.0 Fluorometer (Life Technologies, Carlsbad, CA, USA) and sequenced on one lane using the Illumina HiSeq PE 150 platform.

***Data processing and gene expression analysis***

Adaptor sequences, low-quality reads, and reads containing ploy N were removed from the raw sequencing reads to generate clean reads that were then mapped to the NCBI *Mus musculus* Build 4.0 reference genome. The FPKM (fragments per kilobase per million reads) method was used to eliminate the influence of different gene length and sequencing level on the calculation of gene expression. Transcripts with FDR corrected *P*-value<0.05 and absolute fold-change values ≥1.5 were considered as differentially expressed genes (DEGs), which were then subjected to gene ontology (GO) analysis and KEGG enrichment analysis using DAVID bioinformatics online tools.

***Validation of DEGs by quantitative RT-PCR (qRT-PCR)***

qRT-PCR was performed to validate results from RNA-Seq. Nine DEGs included *SCD2*,* NSDHL*, *Idi1*, *Cyp51*, *Fabp5*,* CNTFR*,* IL6RA*,* EPT1*, and* MVD*. Glyceraldehyde-3-phosphate dehydrogenase (*GAPDH*) was used as the internal reference gene. The information about genes and primers is described in supplemental Table 2. Primers were synthesized by Sangon Biotech Co Ltd (Shanghai, China). qRT-PCR was performed on an ABI 7500 thermocycler (Applied Biosystems, CA, USA) using SYBR Premix Ex Taq reagents (Takara, Dalian, China). qRT-PCR was performed according to Ye* et al*. (19). 

***Statistical analysis***

Data were expressed as the mean±SEM. Differences in means between groups ET and ET-EECP were determined by student’s t-test for unpaired samples using SPSS software (Chicago, IL, USA) (ver. 15.0 for windows). Significance was determined at *P*<0.05.

***Data availability***

The RNA-Seq data discussed in this study have been deposited in NCBI’s Sequence Read Archive under the bioproject PRJNA508551 (SRX5099199, SRX5104371, SRX5104613, SRX5104650, SRX5104737, SRX5104983, SRX5107654, SRX5107655, SRX5107656, SRX5107753, SRX5107752, and SRX5107764 for sample T01-T12, respectively).

## Results

***Influences of EECP on*** ***RLW, BW, hepatic concentrations of TG and TC***

Co-administration of EECP was helpful in ameliorating the injuries resulting from the gavage of ethanol, which was reflected by the decreased serum levels of alanine aminotransferase and aspartate aminotransferase (shown in supplemental Table 3), as well as the improved liver morphology (shown in supplemental Figure 1). 

Compared with mice in group ET, animals in group ET-EECP always had relatively lower RLW. In male animals, the RLWs for groups ET-EECP and ET were 5.39±0.17% and 5.46±0.54%, respectively. However, the difference between them was not significant (*P*=0.083). In female animals, the influence of EECP co-administration on the reduction of RLW was significant. Female mice in group ET-EECP (5.77±0.01%) had significantly lower RLW than female animals in group ET (6.86±0.04%) (*P*<0.001). As to bodyweight (BW), the same gender-associated trend could be observed. The significant BW-reducing influence of EECP was observed only in female animals with group ET-EECP having significantly lower BW (19.50±0.30 g) than group ET (20.53±0.08 g) (*P*=0.030). Male mice in group ET-EECP (22.00±1.19 g) had relatively lower BW than those in group ET (23.90±1.38 g). However, the difference was of no significance (*P*=0.356). Our results indicated that female mice responded more prominently to EECP treatment than male ones with respect to its influences on RLW and BW.

Sex-dependent effects of EECP were also observed in the hepatic concentration of TG. Biochemical assays revealed that co-administration of EECP had a tendency to reduce the hepatic concentration of TG and TC (supplemental Figure 2). However, the influence of EECP on decreasing TG/TC concentration in male ethanol-treated mice was not significant (*P*=0.157 and *P*=0.256 for TG and TC, respectively). Only in female mice, the concentration of hepatic TG was significantly reduced from 0.4051±0.034 to 0.3143±0.003 mmoL per gram protein due to co-administration of EECP (*P*=0.042). Results for all these parameters were shown in supplemental Table 3.

***Transcriptomic profiling of liver tissue***

Results from RNA-Seq showed that approximately 92.81 million clean reads were generated, which provided on average 25,779,462 clean reads per sample. Over 92.31% of these clean reads had a quality score equal or above the Q30 level. More than 95.46% of the reads were mapped to the reference genome, and averagely 93.39% were assigned to the exonic region (Table 1).

***Functional annotation of DEGs and pathway analysis***

Altogether, 665 transcripts were identified as significant DEGs when group ET-EECP was compared with group ET. The most significantly over-represented GO biological processes (BPs) were lipid-related processes, including cholesterol biosynthetic process (13 DEGs, *P*=1.05E-10), lipid metabolic process (44 DEGs, *P*=1.70E-10), steroid metabolic process (17 DEGs, *P*=6.44E-09), steroid biosynthetic process (14 DEGs, *P*=5.12E-08), long-chain fatty acid biosynthetic process (6 DEGs, *P*=3.41E-06), unsaturated fatty acid biosynthetic process (7 DEGs, *P*=3.68E-06), fatty acid biosynthetic process (12 DEGs, *P*=1.85E-05), cholesterol metabolic process (12 DEGs, *P*=1.06E-04), as well as monounsaturated fatty acid biosynthetic process (4 DEGs, *P*=1.20E-04) (as shown in supplemental Table 4). 

**Figure 1:**
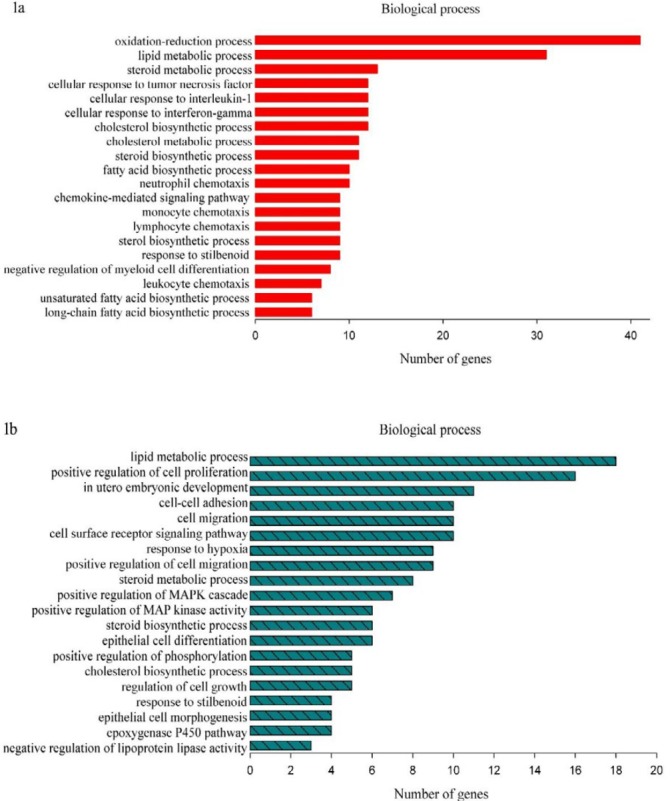
The top 20 gene ontology (GO) biological process (BP) terms with the highest statistical significance

(The vertical axis is the GO BP term, and the horizontal axis is the number of genes. 1a, female mice; 1b, male mice.)

**Figure 2:**
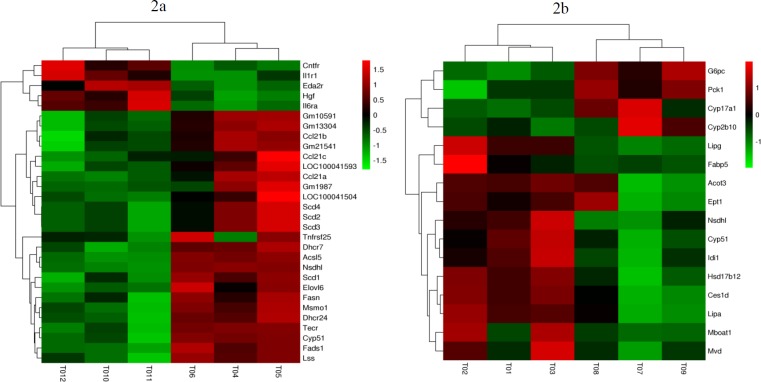
Heatmap showing the expression profile for differentially expressed genes

(Rows are individual samples from group ET (treated with ethanol only) and group ET-EECP (treated with ethanol and co-administered with EECP); Columns are genes with significant expression differences between the two groups. 2a, female mice; 2b, male mice.)

**Figure 3:**
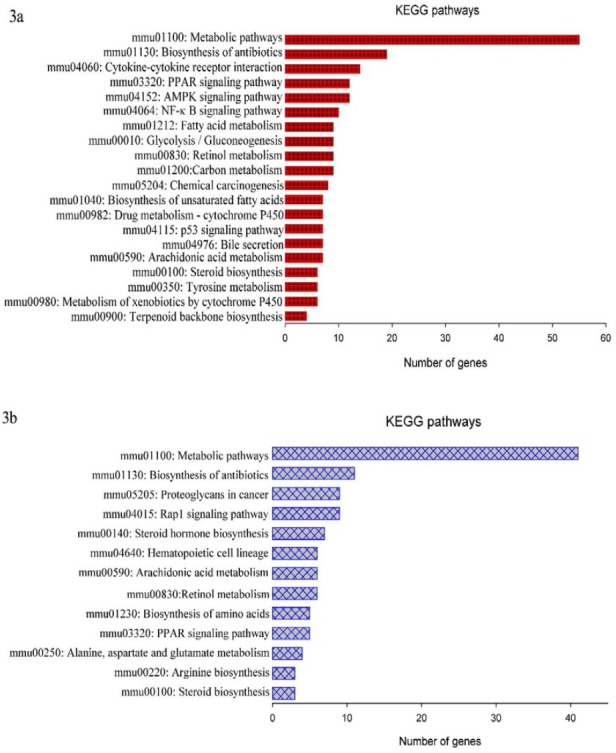
Most significant KEGG pathways

(The vertical axis is the KEGG pathway term, and the horizontal axis is the number of DEGs. 3a, female mice; 3b, male mice.)

**Figure 4:**
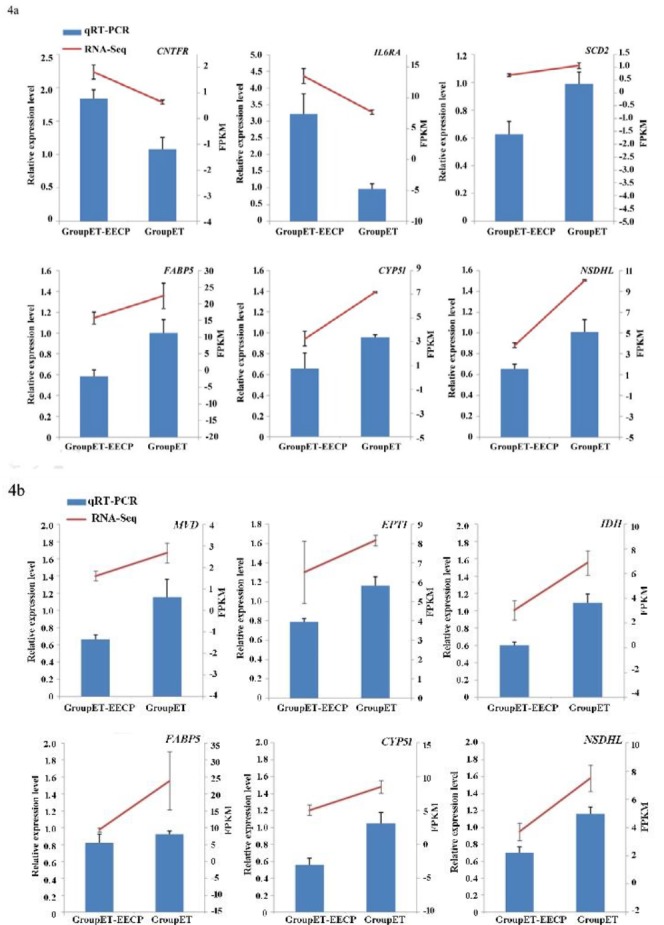
Expression of DEGs validated by qRT-PCR

(Results from RNA-Seq are shown by line graphs on the top and values are shown on the right Y-axis. Results from qRT-PCR are shown by bar graphs on the bottom, and values are shown on the left Y-axis. 4a, female mice; 4b, male mice.)

**Table 1:** General information on the sequencing results

Group	Gender	Sample ID	Clean reads	GC%	%**≥**Q30	Mapped	Exon
ET	Male	T01	24532816	50.11	93.67	95.76%	93.32%
	Male	T02	37881300	49.84	92.31	95.46%	92.78%
	Male	T03	27533253	50.06	93.60	95.76%	94.00%
	Female	T04	22610318	50.36	93.73	96.49%	94.57%
	Female	T05	23873307	49.98	93.53	96.18%	93.67%
	Female	T06	26661805	49.76	93.60	96.23%	93.50%
ET-EECP	Male	T07	23383157	50.15	93.66	96.12%	94.02%
	Male	T08	18337837	50.17	93.55	96.07%	92.17%
	Male	T09	25705446	49.81	93.53	95.58%	92.51%
	Female	T10	28581747	50.12	93.65	96.17%	92.22%
	Female	T11	25297328	51.25	93.56	95.84%	94.09%
	Female	T12	24955239	50.64	93.60	96.19%	93.80%

The most significantly modulated KEGG pathway was the PPAR signaling pathway (*P*=5.09E-07), including 16 DEGs (*SCD1*, *SCD2*, *SCD3*, *SCD4*, *SLC27A1*,* DBI*,* PCK1*, *CD36*, *CYP4A12A*, *CYP7A1*, *CYP8B1*,* FABP1*, *FABP3*,* FABP5*, *ANGPTL4*, and *ACSL5*). Of which, *FABP5*, were significantly down-regulated in both male and female mice in group ET-EECP. Metabolic pathways (85 DEGs, *P*=8.92E-07) and the pathway of biosynthesis of unsaturated fatty acids (9 DEGs, *P*=7.06E-06) ranked the second and the third most significantly modulated pathways, respectively. In the meantime, two lipid-related pathways, steroid biosynthesis (7 DEGs, *P*=6.98E-05) and fatty acid metabolism (10 DEGs, *P*=1.66E-04), were also within the top 10 significantly modulated pathways as shown in supplemental Table 4.

In female mice, a total of 433 genes were identified as significant DEGs when groups ET-EECP and ET were compared. Among these EECP-responsive genes, 152 were up-regulated, and 281 were down-regulated in group ET-EECP. The fold-changes for these DEGs ranged from 1.50 to 4.44. Functional annotation of these DEGs revealed that the top20 significantly over-represented GO biological processes (BPs) (shown in supplemental Table 5) were mainly involved in lipid metabolism (Figure 1a). A general down-regulation was observed in transcripts involved in fatty acid (FA) metabolism pathway (*SCD1*, *SCD2*, *SCD3*, *SCD4*, *FASN*, *FADS1*,* ELOVL6*, *TECR*, and *ACSL5*) and steroid biosynthesis pathway (*CYP51*, *MSMO1*, *DHCR7*, *DHCR24*,* LSS*, *NSDHL*). The other large proportion of these DEGs was categorized into the BP of cytokine signaling. The cytokine-cytokine receptor interaction pathway, containing 15 DEGs (*CCL21A*, *CCL21B*,* CCL21C*, *CNTFR*, *EDA2R*, *HGF*, *IL1R1*,* IL6RA*, *TNFRSF25*, *LOC100041593*, *LOC100041504*, *GM10591*,* GM13304*, *GM1987*, and *GM21541*), was significantly modulated. The differential expression of these 30 DEGs in female mice was displayed by heatmap (Figure 2a), and the significant KEGG pathways were shown in Figure 3a.

In male mice, 279 significant DEGs were identified between groups ET-EECP and ET, which comprised 109 up-regulated genes and 170 down-regulated genes in group ET-EECP. The fold-changes for these DEGs ranged from 1.50 to 7.79. The most significantly enriched GO BP terms (shown in supplemental Table 6) were lipid-related metabolisms, such as steroid and lipid metabolic processes, cholesterol, and steroid biosynthetic processes (Figure 1b). Sixteen genes were involved in the metabolic process of lipids (*ACOT3*,* CES1D*, *CYP17A1*, *CYP2B10*, *CYP51*, *EPT1*, *G6PC*,* HSD17B12*, *IDI1*,* LIPG*, *MBOAT1*, *MVD*, *NSDHL*, *PCK1*,* LIPA*, and* FABP5*). Most of them (12/16) were significantly down-regulated in group ET-EECP except for *CYP17A1*, *CYP2B10*, *G6PC*, and *PCK1*. The cytokine-cytokine receptor pathway was not significantly modulated in male mice. The differential expression of these 16 DEGs in male mice was displayed using a heatmap (Figure 2b), and the significant KEGG pathways were shown in Figure 3b.

As for the nine tested DEGs, the directions of expression level changes were consistent between qRT-PCR and RNA-Seq (Figure 4a and 4b for female and male mice, respectively).

## Discussion

Our results showed that co-administration of EECP had the tendency to reduce RLW, hepatic TG and TC concentrations, and significantly down-regulate the transcriptional expression of genes involved in lipid biosynthesis in ethanol-treated mice (especially the female ones), which was helpful in reducing lipids accumulation in the liver tissue. In female mice, the majority of the DEGs were involved in FA-related BPs, such as FA biosynthesis, FA metabolic process, FA elongation, and long-chain FA biosynthesis. While in male mice, these genes were mainly associated with such BPs as steroid metabolic process and cholesterol biosynthetic process. We suggested that the inhibited lipogenesis due to co-administration of EECP might be achieved through modulation of different BPs in female and male mice.

Another gender-dependent effect of EECP on ethanol-treated mice was that the cytokine-cytokine receptor interaction pathway was significantly modulated only in female mice, which included four down-regulated (*CCL21a*, *CCL21b*,* CCL21c*, and* TNFRSF25*) and five up-regulated annotated genes (*HGF*,* IL-1R1*, *IL6RA, CNTFR*, and *EDA2R*) in group ET-EECP. Elevated expressions of CCL21 (20, 21) and TNFRSF25 (22-24) have been documented in a variety of inflammatory diseases. The significantly down-regulated expression of transcripts *CCL21a/b/c* (three isoforms of *CCL21*) and *TNFRSF25* indicated less severity of inflammation in female mice in group ET-EECP.

Of the five transcripts significantly up-regulated in response to EECP administration in female mice, HGF has been reported to ameliorate liver inflammation during viral hepatitis (25). Its anti-inflammatory features through reducing expression of pro-inflammatory chemokine (26) enable it to limit immune-mediated damage in the liver. IL-1R1 plays a crucial role in inflammation and immune regulation. IL-1R1 knockout mice exhibited higher susceptibility to infection (27, 28) than wild-type mice. As for IL-6RA, the binding of IL-6 to a heterodimeric signaling complex consisting of IL-6RA and the signal-transducing β-subunit glycoprotein 130 (gp130) induces the classic signaling pathway, which is characterized by anti-inflammatory features (29). The significantly elevated expression of *HGF*, *IL-1R1*, and *IL-6RA* in female mice in group ET-EECP might be associated with limited pro-inflammatory effects and would be helpful in the clearance of infection caused by alcohol in the liver tissue. 

Interestingly, the transcriptional expression of *CNTFR*, which encodes the receptor for both cytokine CNTF (30) and the CRLF1 (cytokine receptor-like factor 1)/CLCF1 (cardiotrophin-like cytokine factor 1) dimer (31) also increased significantly in female mice in group ET-EECP. Activation of the CRLF1/CLCF1/CNTFR signaling was reported to slow down the activation of hepatic stellate cells and attenuate the expression of type III collagen in the whole liver (32). It was highly likely that the significantly up-regulated *CNTFR *might be involved in the modulation of fibrosis in ethanol-treated mice.

EDA2R (also known as TNFRSF27) is involved in various signaling pathways, including immune response, inflammation, and carcinogenesis. As a direct p53 (a tumor suppressor gene involved in most human cancers) target, EDA2R exhibited apoptosis-inducing ability (33, 34) and was likely to function as a tumor suppressor (35). The significantly up-regulated expression of *EDA2R *transcript might indicate the enhanced apoptotic signaling in female ethanol-treated mice co-administered with EECP.

Studies in humans have revealed that women are more susceptible to alcoholic toxicity than men (36, 37). Gender-related differences in immune regulation were also documented in ALD (38). Furthermore, existence of remarkable sex differences regarding response to pharmacological treatment (39), distribution of flavonoids metabolites (40), and bioactive effects of various phenolic compounds have been widely described both in animals and human (41). In light of these reported sex-related differences, it was not surprising to detect the gender-related consequences in hepatic transcriptome due to EECP administration in ethanol-treated mice.

Certainly, there are limitations in the present study. The first is the small-size of subjects used in the study. Secondly, there are complex components in EECP, which makes it difficult to clearly explain the exact molecular mechanism underlying the hepatoprotective effects of EECP. Much evidence still needs to be accumulated before EECP can be used in the treatment of ALD in the future.

## Conclusion

Our current results showed that concomitant supplementation of EECP in ethanol-treated mice could decrease the transcriptional expression of genes involved in lipogenesis. In female mice, co-administration of EECP was simultaneously accompanied by significantly modulated expressions of genes associated with the cytokine-cytokine receptor pathway. To our best knowledge, this is the first report that indicates the gender-dependent effects of EECP on ALD. Further investigation of the underlying mechanisms with respect to the effects of flavonoids could be useful for the development of therapeutic interventions to prevent the progress of steatosis to severe forms of liver injury.
